# Linear IgA bullous dermatosis in adults and children: a clinical and immunopathological study of 38 patients

**DOI:** 10.1186/s13023-019-1089-2

**Published:** 2019-05-24

**Authors:** Giovanni Genovese, Luigia Venegoni, Daniele Fanoni, Simona Muratori, Emilio Berti, Angelo Valerio Marzano

**Affiliations:** 1 Dermatology Unit, Fondazione IRCCS Ca’ Granda Ospedale Maggiore Policlinico, Via Pace, 9, 20122 Milan, Italy; 2 Department of Physiopathology and Transplantation, Università degli Studi di Milano, Milan, Italy

**Keywords:** Autoimmune bullous diseases, Linear IgA bullous dermatosis, Adults, Children, Dapsone

## Abstract

**Background:**

Linear IgA bullous dermatosis (LABD) is a rare autoimmune subepithelial vesiculobullous disease due to IgA autoantibodies directed against different antigens of the basement membrane zone (BMZ) of the skin and/or mucosae. It affects mainly preschool-aged children and adults, with only few studies on large series. The aim of this study was to assess possible differences between adults and children regarding clinical presentation, immunopathologic features, management and course of the disease.

**Methods:**

A retrospective review of 38 LABD patients, followed-up from November 2006 to September 2018, was performed.

**Results:**

Of 38 patients, 27 were adults and 11 children. Mean age at diagnosis was 5.4 years and 60.6 years in the pediatric and adult group, respectively. Considering both groups, limbs were the most commonly involved site (73.7%), followed by trunk (55.3%), head (36.8%) and buttocks (13.2%). Interestingly, head (*p* = 0.008), particularly perioral (*p* = 0.001), involvement, as well as “string of pearls” arrangement (*p* = 0.03), were more prevalent in children. Mucosal involvement was seen in 9 (23.7%) patients and was more frequent in children than adults (45.5% vs 14.8%, respectively, *p* = 0.09). Linear IgA deposits along the BMZ were observed in 30 patients (78.9%), while linear/granular IgA deposits in 8 patients (21.1%). Dapsone was the most commonly used drug (78.9%) and complete remission was achieved in most cases (81.6%).

**Conclusions:**

Our epidemiological and clinicopathological findings relative to a large cohort of LABD patients are mostly consistent with the literature data. Interestingly, head, notably perioral, involvement and “string of pearls” arrangement occurred more frequently in the paediatric than adult group. The above clinical parameters may be regarded as diagnostic tools for LABD in children.

**Electronic supplementary material:**

The online version of this article (10.1186/s13023-019-1089-2) contains supplementary material, which is available to authorized users.

## Background

Linear immunoglobulin A (IgA) bullous dermatosis (LABD, ORPHA: 46488) is a rare autoimmune subepithelial vesiculobullous disease caused by IgA autoantibodies directed against different antigens of the basement membrane zone (BMZ) of the skin and/or mucosae [1, 2]. On direct immunofluorescence (DIF), LABD is characterized by linear deposition of IgA along the BMZ, with the possible presence of circulating IgA anti-BMZ antibodies [3]. Both children and adults may be affected, with a bimodal age of onset. In children, LABD typically manifests as tense arciform blisters that may show a “string of pearls” configuration and an erythematous/urticarial base. The preferential sites are reported to be the anogenital area and lower abdomen in children, whilst the trunk and extensor surface of the limbs in adults [1]. The childhood variant, formerly known as chronic bullous disease of the childhood (CBDC), has usually a self-healing course, albeit in few cases it may persist until adulthood [4–10]. Although in the past the adult- and childhood-onset variants were considered as distinct diseases, currently it is widely recognized that these two conditions share the same etiopathogenetic mechanisms and, consequently, have to be unified into a single entity [1]. LABD is a rare disease with an incidence ranging from 0.1 new cases per million population in Germany [11] to 1.45 new cases per million population in Uganda [12]. Mucous membrane involvement, notably oral cavity and conjunctiva, is relatively frequent, potentially leading to scarring sequelae [3, 13–16]. LABD may be idiopathic or due to different triggering factors, including drugs [15–17]. The mainstay of therapy consists of dapsone. In refractory cases, sulfapyridine may be combined or, alternatively, other therapeutic strategies, such as systemic corticosteroids, cyclosporine, colchicine, erythromycin, and intravenous immunoglobulins, may be used [1].

Literature is lacking retrospective studies on LABD from Italy, as only a few case reports have been published [18–29]. Therefore, to expand the knowledge about epidemiological aspects, clinicopathological features, course and management of LABD, we retrospectively studied patients followed-up at a single tertiary care center in Milan (Northern Italy) in order to assess the possible differences between adults and children.

## Materials and methods

### Patients and methods

We retrospectively examined medical records of LABD patients admitted to our outpatient service from November 2006 to September 2018. The diagnosis of LABD was rendered based on both clinical examination and immunopathological criteria [2, 30]. Inclusion criteria were: (i) presence of cutaneous manifestations consisting of tense blisters and/or vesiculobullous lesions and/or erosions, which might show a “string of pearls” configuration, and/or urticaria-like or prurigo-like lesions, possibly associated with oral, conjunctival, nasal or genital mucous membrane involvements consisting of blisters and/or erosions (ii) DIF of perilesional skin showing linear or linear/granular IgA deposition along the BMZ (when concomitant complement fraction 3 [C3c], IgG and/or IgM deposits along the BMZ were observed on DIF, the diagnosis of LABD was confirmed only if IgA deposits were predominant). Indirect immunofluorescence (IIF) with salt-split skin was performed to detect IgA and IgG deposits only in selected cases when the diagnosis was uncertain. Patients with predominant/exclusive mucosal manifestations were diagnosed as having mucous membrane pemphigoid (MMP) based on the diagnostic criteria set up in the first international consensus on MMP [31]; therefore, they were excluded from the study. In all patients with linear/granular IgA deposits on DIF, a possible diagnosis of dermatitis herpetiformis (DH) was ruled out on the basis of the negativity of serum IgA antibodies against both tissue transglutaminase and endomysium. Clinical parameters collected included sex, age at disease onset, comorbidities, pre-biopsy differential diagnosis, involved sites and clinical presentation, management strategies, and outcome at the last follow-up. Drug-induction was assessed by means of Naranjo score (NS), with an at least probable score (> 4) as the threshold to diagnose drug-induced LABD [32]. “Complete remission” (CR) was defined as the absence of new and/or established lesions for at least 2 months without or with minimal therapy. “Minimal therapy” was considered as less than or equal to 0.2 mg/kg/day of dapsone and/or 0.1 mg/kg/day of prednisone (or the equivalent) and/or minimal adjuvant or maintenance therapy. “Partial remission” (PR) was defined as the presence of transient (healing within a week) new lesions without or with minimal therapy, as defined above. No response (NR) was defined as the presence of persistent (not healing within a week) new lesions despite therapy (Additional file 1: Table S1). Relapses were defined as the reappearance of LABD manifestations in patients who showed an at least 4-month duration CR.

### Statistical analysis

Categorical variables are reported as number (percentage), and continuous variables as mean (range). Data analysis was performed with Fisher’s exact test, as appropriate, using GraphPad Prism version 6.0 (GraphPad Software, Inc., San Diego, CA). Statistical significance was defined as *p* ≤ 0.05.

## Results

### Patients and clinical findings

**Fig. 1:**
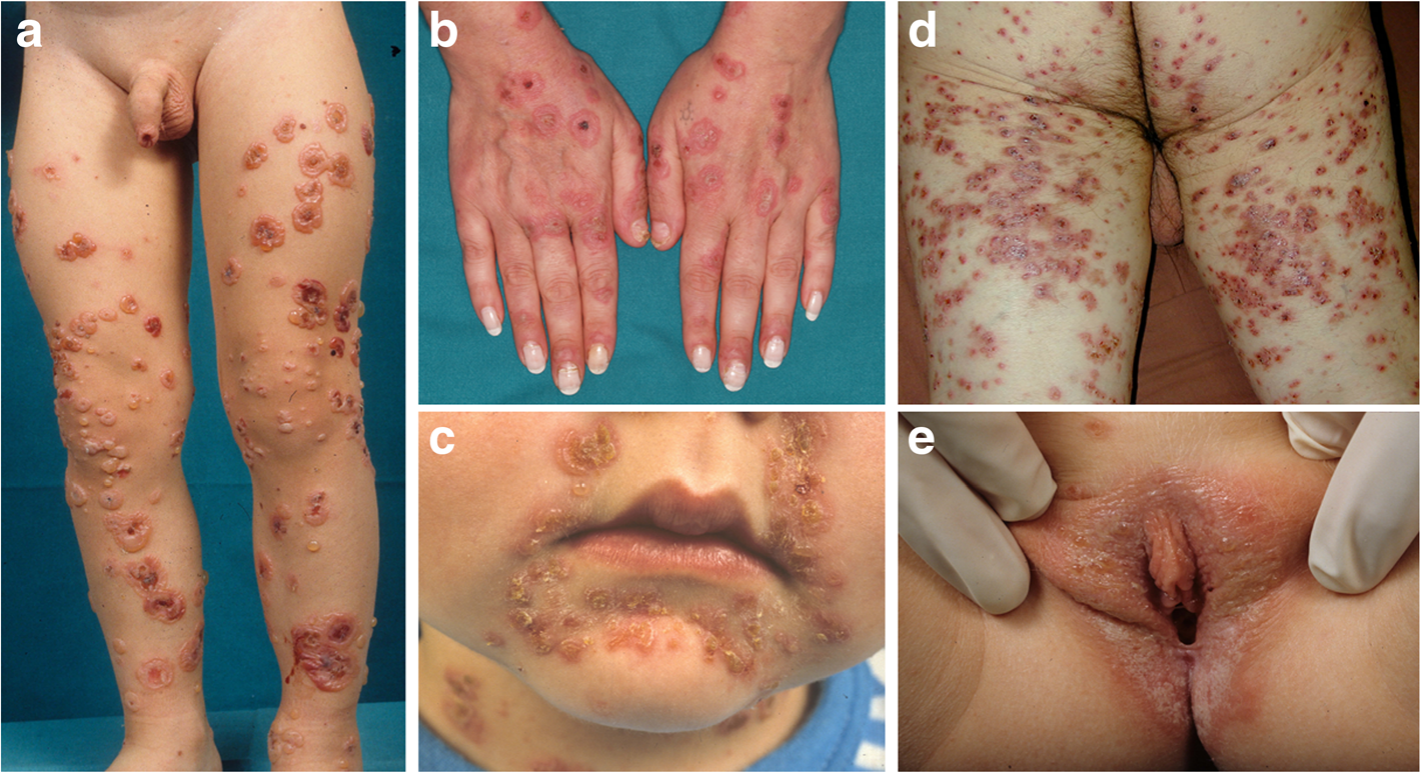
Linear IgA bullous dermatosis. **a** Widespread vesiculobullous eruption on the lower limbs with elements in a “string of pearls” arrangement; (**b**) targetoid vesicular lesions on erythematous skin involving the dorsa of the hands; (**c**) blisters with a “string of pearls” configuration and crusts in the perioral area; (**d**) erythematous, vesicular lesions partially eroded on the posterior aspects of both thighs; (**e**) vesicles involving the vulvar area in a child

Detailed clinical and laboratory data of the 38 collected patients are shown in Additional file 1: Table S1 included in the manuscript as additional file. They had an average age at diagnosis of 45.7 years (range 0.9–93 years). In the 11 patients (28.9%) diagnosed before 16 years old, the mean age at diagnosis was 5.4 years, while in the adult group it was 60.6 years. The overall male to female ratio was 1.2. It was lower in adults (0.9) than in children (2.7), even though, as shown in Table 1, there were no statistically significant differences in sex preponderance between adults and children. Concerning comorbidities, four patients had a history of neoplasm, two were affected by ulcerative colitis and one by coeliac disease. This last patient displayed linear/granular IgA deposits, making it mandatory to rule out DH. Thus, we performed salt-split skin IIF that showed linear IgA deposits along the epidermal side of the BMZ, consistent with LABD. At the time of diagnosis, bullous and/or vesiculobullous elements on erythematous (Fig. 1b and d) or non-inflamed skin represented the most commonly observed (*n* = 35; 92.1%) skin lesions. The “string of pearls” arrangement (Fig. 1a and c) was described in 8 patients (21.1%) and resulted significantly more frequent in children than in adults (45.5% vs 11.1%; *p* = 0.03), as shown in Table 1. In a single case, only urticaria-like lesions were present, while in two cases crusts, erosions and excoriated lesions were the only cutaneous findings. Skin lesions were located on the limbs (*n* = 28: 73.7%), trunk (*n* = 21; 55.3%), head (*n* = 14; 36.8%) and buttocks (*n* = 5; 13.2%). Head involvement was more common in children than adults (72.7% vs 22.2%; *p* = 0.008) and, when considering only the perioral area, the statistical significance increased (54.5% vs 3.7%; *p* = 0.001). Mucous membrane involvement was observed in 9 (23.7%) patients and the most frequent mucosal localisation was oral cavity (15.8%), followed by genital (7.8%) (Fig. 1e), conjunctival (5.2%) and nasal cavity (2.6%) involvements. Mucosae were more frequently involved in children than adults (45.5% vs 14.8%, respectively; *p* = 0.09) and, interestingly, all patients with genital involvement (*n* = 3) were children. Pre-biopsy diagnostic hypothesis was correct in 7 cases (18.4%), while bullous pemphigoid (BP) (*n* = 10; 26.3%) and bullous impetigo (*n* = 5; 13.2%) represented the most frequent misdiagnoses. All but two cases were idiopathic; the two probably drug-induced cases were triggered by amoxicillin/clavulanic acid (NS = 6) and clarithromycin (NS = 5), with a latency time from the intake of 1 and 3 weeks, respectively. Losartan (NS = 2), oxcarbazepine (NS = 3) and chlorambucil (NS = 2) were regarded as possible culprit drugs in three other cases.

**Table 1:** Clinical and laboratory characteristic of adults and children with LABD

Characteristic	Adults (*n* = 27)	Children (*n* = 11)	*p*-value
Male patients	13 (48.1)	8 (72.7)	0.28
Mean age at diagnosis (years)	60.6	5.4	–
“String of pearls” pattern	3 (11.1)	5 (45.5)	0.03^*^
Mucosal involvement	4 (14.8)	5 (45.5)	0.09
Head involvement (excluded mucosae)	6 (22.2)	8 (72.7)	0.008^*^
Perioral involvement	1 (3.7)	6 (54.5)	0.001^*^
Scalp involvement	4 (14.8)	0	0.3
Limbs	20 (74.1)	8 (72.7)	0.93
Trunk	15 (55.6)	6 (54.5)	1
Buttocks	4 (14.8)	1 (9.1)	1
Drug-induction	1 (3.7)	1 (9.1)	0.5
Eosinophils on histopathology	17 (62.7)	5 (45.5)	0.47
Linear IgA along the BMZ at DIF	21 (77.8)	9 (81.8)	1
Linear/granular IgA along the BMZ at DIF	6 (22.2)	2 (9.1)	1
C3c deposits at DIF	7 (25.9)	4 (36.4)	0.7
Salt-split skin IIF positivity for linear IgA^a^	13 (43.5)	6 (85.7)	0.21
CR^b^	21 (84)	10 (90.9)	1
PR^b^	2 (8)	1 (9.1)	1
NR^b^	2 (8)	0	1
Relapses^b^	4 (16)	1 (9.1)	1

Values are expressed as n (%); C3c, complement fraction 3; CR, complete remission; DIF, direct immunofluorescence; IIF, indirect immunofluorescence; NR, no response; PR: partial remission; ^a^, performed in only 30 patients (23 adults and 7 children); ^b^, 2 patients lost at follow-up; *, statistically significant

### Histology and immunofluorescence results

Histological examination on skin or mucous biopsies as well as DIF examination were routinely performed in all patients. On histopathological assessment, all cases revealed subepidermal blisters associated with dermal infiltrate of neutrophils. In 22 cases (57.9%), eosinophils were observed in association with neutrophils in the upper dermis, without statistically significant differences between adults and children (*p* = 0.47). Neutrophil microabscesses at the tip of the dermal papillae were seen in three cases (7.9%).

On DIF, linear IgA deposits along the BMZ were observed in 30 patients (78.9%), while linear/granular IgA deposits were detected in 8 patients (21.1%). As shown in Table 1, no statistically significant differences were observed between adults and children in terms of positivity for linear or linear/granular IgA deposits. Linear deposits of IgA alone - with neither Ig class other than IgA nor complement - were observed in 23 cases, while linear/granular deposits of IgA alone were observed in three cases. Among the 12 cases showing deposits of Ig other than IgA or complement in addition to IgA, 11 patients had C3c, 6 had IgM and 3 had IgG. Salt-split human skin IIF was performed in 30 patients and was positive in 19 out of 30 cases (63.3%), all showing linear IgA deposits along the epidermal side of the BMZ. All cases were negative for IgG deposits on salt-split human skin IIF. Children had a higher, albeit not statistically significant, positivity rate of salt-split human skin IIF compared to adults (85.7% vs 43.5%; *p* = 0.21).

### Treatment and follow-up

All patients except two self-resolving cases received systemic therapy, combined with topical treatment in some cases. Dapsone, administered to 30 patients (78.9%), with a mean starting dosage of 0.5 mg/kg per day, was the most common treatment option. Median duration of dapsone treatment was 26.2 months. We observed dapsone-induced methemoglobinemia only in a patient, who was switched to oral methylprednisolone and consequently showed a CR. Systemic corticosteroids (prednisone, oral and intravenous methylprednisolone, and deflazacort) were given in concomitance with, preceding or following dapsone in 22 cases (57.9%), while in 5 cases (13.2%) they represented a stand-alone therapy. In a case resistant to corticosteroid monotherapy, a combination of methotrexate and oral methylprednisolone induced a CR. Other treatment options for steroid-refractory cases are reported in Additional file 1: Table S1. Topical steroids were given to 6 patients, with clobetasol propionate being the most commonly used agent (*n* = 4; 10.5%). Cyclosporine eye drops were administered in two patients with ocular involvement. One adult patient with a drug-induced form and one pediatric patient received neither systemic nor topical treatments owing to a self-limiting course, with a follow-up time of 16 months in both cases. The mean follow-up time was 30.7 months. CR was achieved in most cases (*n* = 31; 81.6%), while PR in three patients. Only two cases were refractory to multiple drugs, showing NR (Additional file 1: Table S1). Scarring sequelae were observed in two cases showing aggressive conjunctival involvement. Relapses were observed in five patients (adults, *n* = 4; children, *n* = 1), with no statistically significant differences between adults and children. All patients with relapsed disease were not taking any medication for their LABD at the moment of the relapse. The mean time from drug withdrawal to relapse was 10 months. All patients who experienced a relapse re-achieved a CR after treatment reintroduction. As shown in Table 1, no statistically significant differences were found between the adult and pediatric group in terms of response to treatment.

## Discussion

We present the largest case-series of Italian patients with LABD and compare our findings with data reported in the literature (Additional file 2: Table S2). Our results confirmed the bimodal age at onset of this disease [1], with two different groups represented by children and adults. Although an overall similar prevalence in males and females was outlined, stratifying the population in terms of age, our data revealed a preponderance of the disease, albeit not statistically significant, in male children, as already observed by other authors [7–9].

Regarding clinical manifestations, of note, in our study head, particularly perioral, involvements were significantly more common in the pediatric than adult group (72.7% vs 22.2%; *p* = 0.008 and 54.5% vs 3.7%; *p* = 0.001, respectively). This is in line with literature data reporting the head and face more commonly involved in children than adults [6]. Furthermore, the “string of pearls” arrangement was significantly more frequent in children than adults (*p* = 0.03), similarly to the findings of Jabłońska et al. [5], with an overall prevalence of 21%. Consistent with data reporting high prevalences of genital involvement in the pediatric population [5], all patients with genital involvement were children.

Gottlieb et al. [14] showed that 60% of a case series of 72 adults with LABD had mucous membrane involvement, suggesting that the term “linear IgA disease” might be more suitable than “linear IgA dermatosis”. On the other hand, data about mucous membrane involvement in children are controversial, since a study by Wojnarowska et al. [6] disclosed a prevalence of mucosal involvement of 64% in 25 LABD children, but more recent case series revealed lower prevalences [9, 10, 33]. In our study, mucosal involvement resulted less common than that reported by Wojnarowska et al. [6] and it was more frequent in children than adults (45.5% vs 14.8%, respectively), albeit without statistically significant difference.

We regarded drugs as a probable or possible cause of LABD in 13.2% of our cohort, a lower prevalence as compared to the study of Lings et al., which identified possibly/probably drug-induced LABD in 6 out of 23 (26.1%) cases [34]. Although both Chanal et al. [16] and Garel et al. [15] concluded in two independent retrospective studies that drug-induced LABD might be more severe and mimic toxic epidermal necrolysis (TEN), both our drug-induced cases lacked TEN-like features and achieved a CR upon treatment.

The increasingly described association of LABD with inflammatory bowel diseases [35–37] was confirmed by our study, showing an ulcerative colitis prevalence of 5.6%, much higher than 0.12% estimated in the Italian general population [38]. Immune activation secondary to the exposition of multiple intestinal epithelial antigens, included BP180, in patients with coexistence of LABD and inflammatory bowel diseases has been hypothesized as a possible pathomechanism eliciting blister formation [35].

Approximately 15% of the adult patients had a history of cancer. Although malignancies, especially of lymphoreticular origin, have been described in association with LABD, [39] in all our cases the lack of a close temporal relationship between the onset of LABD and the underlying neoplasm made it unlikely the hypothesis of a paraneoplastic disease.

Linear IgA deposition along the BMZ with neither Ig class other than IgA nor complement resulted the most frequent pattern on DIF in our case series. In our study, patients with linear IgG deposits in addition to linear IgA deposits were considered as a subgroup of LABD. Indeed, Ohata et al. [3] suggested linear IgA/IgG bullous dermatosis as part of a spectrum of diseases ranging from LABD to BP.

IIF on human skin has been reported to be positive for IgA in just over 80% of children and approximately 40% of adults [40] and salt-split skin technique demonstrated an increase in the sensitivity of IIF [3, 14, 40]. In our study, salt-split skin IIF resulted positive in approximately 60% of patients and, in line with the results of Willsteed et al. [40], a higher, albeit not statistically significant, rate of positivity of salt-split skin IIF was observed in children as compared to adults (85.7% vs 43.5%, respectively). Moreover, all our cases showed an epidermal binding. Although IgA deposits on salt-split skin are predominantly detected on the epidermal side [40], dermal binding has been reported in 8% [3] and 17% [41] of large case series. In the study by Ohata et al. [3], binding of IgA autoantibodies to both sides of the split was observed only in 7% of patients.

Most frequent and successful treatment was dapsone, given both as a stand-alone therapy and combined with systemic steroids. In our comparison of the adult and children group, we failed to identify any differences in terms of response to treatment. As compared to the study of Gottlieb et al., [14] higher CR and lower relapse rates were observed in our patients. These differences might be due to a combination of the following aspects: (i) only adults, usually showing a more aggressive and relapsing course, have been collected in the study of Gottlieb et al.; (ii) as opposed to our study, cases with predominant mucous membrane involvement, also showing a more refractory behaviour, have been included; (iii) topical corticosteroids were the first-choice treatment in 40% of their patients, while almost all our patients received systemic treatments as first-line.

The main limitation of the present study was its retrospective nature, which, however, depended on the rarity of the disease. Furthermore, a direct comparison between the two groups might have been biased by the fact that adults were more numerous than children in the study population. An additional limitation was represented by the fact that immunoblotting and enzyme-linked immunoassay for BP180, BP230 and/or collagen VII were not performed. Therefore, it cannot be stated with certainty whether cases of sublamina densa-type LABD and IgA-epidermolysis bullosa acquisita, whose common autoantigen is collagen VII, [42–44] have been missed. Furthermore, establishing an accurate diagnosis of LABD may be difficult owing to the absence of unanimous consensus on diagnostic criteria. [45] In this regard, we ruled out from our study patients with linear or linear/granular IgA deposits on DIF and predominant/exclusive mucosal involvement, who, based on the first consensus conference on MMP, were classified as having IgA-MMP. [31, 46] Although our criteria may have been strict and are not fully in line with those adopted in other large series on LABD [3, 14], we believe that they are clear and have minimal risk of including MMP patients.

## Conclusion

In summary, the results of this retrospective study conducted on a cohort of 38 Italian LABD patients diagnosed and followed-up in a single center confirmed the biphasic age at onset of the disease and showed a higher prevalence of head, particularly perioral, and mucosal involvements as well as a more frequent “string of pearls” arrangement in children than adults. Thus, these clinical parameters may be a diagnostic tool for LABD in children.

## Additional files

Additional file 1: **Table S1.** Demographic data, clinical features, diagnostic findings, treatments and outcomes of 38 linear IgA bullous dermatosis patients. (DOCX 37 kb)

Additional file 2: **Table S2.** Summary of the main studies on linear IgA bullous dermatosis including study design, patients’ number, clinical features and laboratory findings. (DOCX 25 kb)

## Abbreviations

BMZ: basement membrane zone
BP: bullous pemphigoid
C3c: complement fraction 3
CBDC: chronic bullous disease of the childhood
CR: complete remission
DH: dermatitis herpetiformis
DIF: direct immunofluorescence
Ig: immunoglobulin
IIF: indirect immunofluorescence
LABD: linear bullous IgA dermatosis
MMP: mucous membrane pemphigoid
NR: no response
NS: Naranjo score
PR: partial remission
TEN: toxic epidermal necrolysis
