# Supplementary material for: Linear IgA bullous dermatosis in adults and children: a clinical and immunopathological study of 38 patients
Source: Orphanet J Rare Dis. 2019 May 24;14:115. doi: 10.1186/s13023-019-1089-2 (PMC6534856; doi:10.1186/s13023-019-1089-2)
Supplement: Supplementary file 1 — Table S1. Demographic data, clinical features, diagnostic findings, treatments and outcomes of 38 linear IgA bullous dermatosis patients. (DOCX 37 kb) [file 13023_2019_1089_MOESM1_ESM.docx]

ADDITIONAL FILE

TABLE S1. Demographic data, clinical features, diagnostic findings, treatments and outcomes of 38 linear IgA bullous dermatosis patients

| **Patient N°** | **Age (yrs) / sex** | **Comorbidities** | **Histology** | **Clinical features** | **DIF** | **Salt-split skin IIF** | **Treatment** | **Response (follow-up time, months)** | **Drug-induction (NS)** | **Pre-biopsy diagnosis** |
| --- | --- | --- | --- | --- | --- | --- | --- | --- | --- | --- |
| 1 | F/34 | None | Subepidermal blister, neutrophils, eosinophils | Vesiculobullous eruption/SOF/inframammary and retroauricular folds and scalp | Linear IgA and C3c deposits along the BMZ | Linear IgA deposits (epidermal side of the BMZ) | Dapsone + intravenous methylprednisolone, plasmapheresis, cyclophosphamide, mycophenolate mofetil, rituximab | NR (132) | None | LABD |
| 2 | M/29 | None | Subepidermal blister, neutrophils | Vesiculobullous eruption/periumbilical region, perioral area | Linear IgA deposits along the BMZ | Negative | Dapsone | CR (37) | None | LABD |
| 3 | F/74 | OA | Subepidermal blister, neutrophils | Erosive-crusted and excoriated lesions/trunk | Linear IgA deposits along the BMZ | Negative | Prednisone, methotrexate + oral methylprednisolone | CR (97), relapse at month 52 | None | BP |
| 4 | F/35 | None | Subepidermal blister, neutrophils, eosinophils | Blisters/SOF/trunk, upper limbs | Linear IgA deposits along the BMZ | Negative | Dapsone | CR (35) | None | Pemphigus herpetiformis |
| 5 | M/72 | None | Subepidermal blister, neutrophils, eosinophils | Blisters/scalp, upper limbs | Linear IgA and granular C3c deposits along the BMZ | ND | Dapsone + prednisone | CR (62), relapse at month 41 | None | EBA |
| 6 | F/80 | CLL | Subepidermal blister, neutrophils, neutrophil microabscesses, eosinophils | Blisters/trunk, upper and lower limbs | Linear/granular IgA deposits along the BMZ | Negative | Deflazacort + topical clobetasol | CR (4) | Chlorambucil (2) | BP |
| 7 | F/51 | None | Subepidermal blister, neutrophils, eosinophils | Blisters/inframammary folds | Linear IgA deposits along the BMZ | Linear IgA deposits (epidermal side of the BMZ) | Dapsone + azathioprine + prednisone | CR (18) | None | BP |
| 8 | M/75 | AH, CAD, CKD | Subepidermal blister, neutrophils | Blisters and erosions/ upper limbs, buttocks; erosions/oral cavity | Linear/granular IgA and granular C3c deposits along the BMZ | ND | Dapsone, prednisone | CR (8) | None | BP |
| 9 | M/68 | CD, nodular prurigo | Subepidermal blister, neutrophils, eosinophils | Urticarial lesions/elbows, knees, popliteal fossae, inguinal folds | Linear/granular IgA deposits and C3c cytoid bodies along the BMZ | Linear IgA deposits (epidermal side of the BMZ) | Dapsone, oral methylprednisolone | CR (5) | None | DH |
| 10 | F/41 | None | Subepidermal blister, neutrophils, eosinophils | Vesiculobullous eruption/ upper limbs | Linear/granular IgA, C3c and IgM deposits along the BMZ; | Negative | Prednisone | CR (69) | None | Bullous insect bite reaction |
| 11 | F/75 | None | Subepidermal blister, neutrophils, eosinophils | Vesiculobullous eruption/trunk, upper limbs | Linear IgA deposits along the BMZ | Negative | Prednisone | CR (10) | None | LABD |
| 12 | M/53 | GERD, IV  AH, DVT | Subepidermal blister, neutrophils, eosinophils | Blisters/upper limbs, inguinal folds, trunk, buttocks; erosions/oral cavity | Linear/granular IgA deposits along the BMZ | ND | Dapsone + prednisone, methotrexate | CR (71), relapse at month 45 | None | LABD |
| 13 | M/54 | None | Subepidermal blister, neutrophils | Vesiculobullous, erosive and excoriated lesions/upper and lower limbs | Linear IgA deposits along the BMZ | Negative | Prednisone, dapsone + topical methylprednisolone | CR (50) | None | BP vs eczema |
| 14 | F/64 | None | Subepidermal blister, neutrophils, eosinophils | Blisters/upper and lower limbs; erosions and symblepharon/ corneal-conjunctival surface of both eyes; erosions/gingival mucosa | Linear IgA deposits along the BMZ | Linear IgA deposits (epidermal side of the BMZ) | Dapsone + prednisone; cyclosporine eye drops | PR (62) | None | CP |
| 15 | F/67 | AD, epilepsy, AH | Subepidermal blister, neutrophils, neutrophil microabscesses | Vesiculobullous eruption/ SOF/ scalp trunk, buttocks | Linear IgA deposits along the BMZ | Negative | Dapsone + prednisone + topical clobetasol | CR (20) | Oxcarbazepine (3) | BP |
| 16 | F/66 | AH, CAD | Subepidermal blister, neutrophils, eosinophils | Vesicles on erythematous base/trunk, lower limbs | Linear IgA and linear/granular C3c deposits along the BMZ | Linear IgA deposits (epidermal side of the BMZ) | Dapsone + prednisone | CR (41), relapse at month 33 | None | Eczema |
| 17 | F/69 | None | Subepidermal blister, neutrophils | Blisters/trunk | Linear IgA and C3c and linear/granular IgM deposits along the BMZ | ND | Dapsone, prednisone | CR (39) | None | Herpes zoster, pemphigus vulgaris |
| 18 | M/51 | UC, osteoporosis | Subepidermal blister, neutrophils, eosinophils | Erosions and crusts/upper and lower limbs, trunk, scalp; blisters/trunk, lower limbs | Linear IgA deposits along the BMZ | Linear IgA deposits (epidermal side of the BMZ) | Dapsone + prednisone, azathioprine, cyclophosphamide, mycophenolate mofetil, rituximab, methotrexate | NR (38) | None | Eczema |
| 19 | F/61 | None | Subepidermal blister, neutrophils | Vesicles and crusted lesions/ upper and lower limbs | Linear IgA deposits along the BMZ | Linear IgA deposits (epidermal side of the BMZ) | Azathioprine + prednisone + topical clobetasol, dapsone | CR (36) | None | Herpes zoster |
| 20 | F/82 | AH, GERD | Subepidermal blister, neutrophils, eosinophils | Blisters/lower limbs | Linear IgA deposits along the BMZ | Linear IgA deposits (epidermal side of the BMZ) | Dapsone | N/A | None | BP |
| 21 | F/71 | Breast cancer | Subepidermal blister, neutrophils | Urticarial lesions and blisters/upper limbs (>> back of the hands) | Linear/granular IgA deposits along the BMZ | Linear IgA deposits (epidermal side of the BMZ) | Dapsone | CR (27) | None | Chronic urticaria |
| 22 | M/43 | Renal transplant for CKD, β-thalassemia minor | Subepidermal blister, neutrophils, eosinophils | Vesiculobullous lesions/trunk; crusted lesions on erythematous base/ face (beard area) | Linear IgA deposits along the BMZ | Linear IgA deposits (epidermal side of the BMZ) | Prednisone, dapsone + topical clobetasol | CR (13) | None | Sycosis vulgaris |
| 23 | M/93 | AH, ischemic stroke | Subepidermal blister, neutrophils, neutrophil microabscesses, eosinophils | Blisters on erythematous base/ trunk, upper and lower limbs | Linear IgA deposits along the BMZ; granular IgA perivascular deposits | Linear IgA deposits (epidermal side of the BMZ) | Prednisone | CR (5) | Amoxicillin/clavulanic acid (6) | BP |
| 24 | M/81 | Prostate cancer, AH, chronic HCV infection | Subepidermal blister, neutrophils, eosinophils | Blisters on urticarial base/lower and upper limbs, buttocks | Linear IgA deposits along the BMZ | Negative | Dapsone + prednisone | CR (17) | Losartan (2) | Erythema multiforme vs chronic urticaria |
| 25 | M/46 | None | Subepidermal blister, neutrophils | Blisters/trunk, upper and lower limbs | Linear IgA deposits along the BMZ | Linear IgA deposits (epidermal side of the BMZ) | Prednisone | N/A | None | BP |
| 26 | M/76 | MEN 1 | Subepidermal blister, neutrophils | Blisters/upper and lower limbs; erosions and ulcerations/oral cavity, conjunctiva of both eyes | Linear IgA deposits along the BMZ | Linear IgA deposits (epidermal side of the BMZ) | Rituximab + intravenous methylprednisolone + dapsone, cyclosporine eye drops | PR (17) | None | CP |
| 27 | M/26 | None | Subepidermal blister, neutrophils, eosinophils | Vesiculobullous eruption/trunk, ankles | Linear IgA deposits along the BMZ | Negative | Self-resolution after drug withdrawal | CR (16) | Clarithromycin (5) | BP |
| 28 | F/6 | None | Subepidermal blister, neutrophils, eosinophils | Vesiculobullous eruption on erythematous skin/ trunk, ears, lower and upper limbs; vesicles and erosions/genital mucosa, | Linear IgA deposits along the BMZ | ND | Dapsone + oral betamethasone | CR (12) | None | Bullous impetigo |
| 29 | M/7 | None | Subepidermal blister, neutrophils | Blisters/face (>> perioral area), oral cavity, nasal cavity | Linear IgA deposits along the BMZ | Linear IgA deposits (epidermal side of the BMZ) | Self-resolution without therapy | CR (16) | None | Herpes simplex virus infection |
| 30 | F/4 | None | Subepidermal blister, neutrophils, eosinophils | Vesicles on erythematous skin/perioral and periumbilical area; blisters/trunk; erosions and vesicles/genital mucosa, perianal area | Linear IgA deposits along the BMZ | ND | Dapsone | CR (6) | None | Atopic dermatitis |
| 31 | M/0.9 | None | Subepidermal blister, neutrophils | Vesicles on erythematous skin/ face, trunk | Linear/granular IgA and linear IgM and C3c deposits along the BMZ | Linear IgA deposits (epidermal side of the BMZ) | Dapsone + prednisone | CR (5) | None | LABD |
| 32 | M/14 | UC | Subepidermal blister, neutrophils | Blisters/trunk, upper and lower limbs | Linear IgA deposits along the BMZ | Linear IgA deposits (epidermal side of the BMZ) | Dapsone + prednisone | CR (8) | None | Chickenpox |
| 33 | M/3 | None | Subepidermal blister, neutrophils, eosinophils | Blisters/SOF/lower and upper limbs, face (>> perioral area) | Linear IgA deposits and IgM cytoid bodies along the BMZ; granular C3c deposits at dermal tips | Linear IgA deposits (epidermal side of the BMZ) | Dapsone | CR (22) | None | Bullous impetigo |
| 34 | M/2 | None | Subepidermal blister, neutrophils | Vesiculobullous eruption/SOF/perioral area, upper limbs, ears; erosions/genital mucosa | Linear IgA deposits along the BMZ | ND | Dapsone | CR (19) | None | Bullous impetigo |
| 35 | F/10 | None | Subepidermal blister, neutrophils | Vesicles and crusted lesions on erythematous skin/trunk, lower limbs; ulceration/oral cavity | Linear/granular IgA, IgG and C3c deposits along the BMZ | ND | Dapsone, prednisone | PR (26) | None | Bullous impetigo vs ACD |
| 36 | M/1 | None | Subepidermal blister, neutrophils, eosinophils | Vesiculobullous eruption on erythematous skin/SOF/ lower and upper limbs, face (>> perioral area) | Linear IgA deposits and IgM cytoid bodies along the BMZ | Linear IgA deposits (epidermal side of the BMZ) | Dapsone | CR (32), relapse at month 26 | None | LABD |
| 37 | M/4 | None | Subepidermal blister, neutrophils, eosinophils | Blisters/SOF/ face (>>perioral area), ears, lower limbs, elbows, palmoplantar regions, buttocks | Linear IgA, C3c, IgG and IgM deposits along the MBZ | Negative | Dapsone + prednisone | CR (25) | None | Bullous impetigo |
| 38 | M/3 | None | Subepidermal blister, neutrophils | Blisters/SOF/Back of the hands, lower limbs, trunk | Linear IgA deposits along the BMZ | Linear IgA deposits (epidermal side of the BMZ) | Dapsone + prednisone + topical gentamycin/  betamethasone | CR (28) | None | LABD |

ACD, allergic contact dermatitis; AD, Alzheimer’s disease; AH, arterial hypertension; BP, bullous pemphigoid; BMZ basement membrane zone; CD, coeliac disease; C3c, complement fraction 3; CAD, coronary artery disease; CLL, chronic lymphocytic leukemia; CKD, chronic kidney disease; CP, cicatricial pemphigoid; CR, complete remission; DH, dermatitis herpetiformis; DVT, deep vein thrombosis; EBA, epidermolysis bullosa acquisita; GERD, gastroesophageal reflux disease; IV, ichthyosis vulgaris; MEN1, multiple endocrine neoplasia type 1; N/A, not available; ND, not done; NR, no response; NS, Naranjo score; OA, osteoarthrosis; PR, partial remission; SOF, “string of pearls” pattern; UC, ulcerative colitis

TABLE S2. Summary of the main studies on linear IgA bullous dermatosis including study design, patients’ number, clinical features and laboratory findings

| **First author (year)** | **Design** | **Population (n)** | **Conclusions** | **Ref.** |
| --- | --- | --- | --- | --- |
| Chanal (2013) | Retrospective study | 28 [16 with spontaneous form and 12 with drug-induced LABD] | Drug-induced LABD was more severe than the idiopathic variant, with Nikolsky sign and large erosions resembling those of toxic epidermal necrolysis | 16 |
| Garel (2019) | Retrospective study | 69 [with drug-induced LABD] | Drug-induced LABD tends to be associated with severe clinical manifestations that may sometimes mimic toxic epidermal necrolysis. Vancomycin was suspected to be the culprit drug in > 50% of the cases and was associated with more severe course. | 15 |
| Gottlieb (2017) | Retrospective study | 72 [adults] | In adults, LABD had an increased risk for chronic evolution in patients less than 70 years old and with mucosal involvement | 14 |
| Kanwar (2004) | Retrospective study | 12 [children] | In India, LABD tends to present with a generalized eruption. Minimal mucosal involvement and excellent response to dapsone monotherapy were observed. | 6 |
| Kelly (1988) | Clinicopathological study | 10 mucosal LABD [10 with oral and 6 with conjunctival involvement] | All patients with clinical oral disease had linear IgA deposits along the BMZ of oral mucosa, whilst no linear IgA could be demonstrated in conjunctival biopsies. Conjunctiva provided a good substrate for IIF and revealed that 50% of all patients had circulating anti-BMZ IgA. | 13 |
| Kenani (2009) | Retrospective study | 25 [children] | Most patients experienced a prompt response to dapsone, which was the most commonly used drug. Minimal mucosal involvement was observed. | 9 |
| Kharfi (2010) | Retrospective study | 47 [children] | Most patients presented with a generalized eruption. Mucosal involvement was noticed in few patients. Most patients, in addition to IgA, showed evidence of IgG, IgM or complement deposits on DIF. Dapsone was the first-line therapy in 70% of the cases, leading to complete remission in 8 cases. | 8 |
| Kong (2015) | Retrospective study | 20 children with AIBDs [5 with LABD] | In Singapore, LABD is the most common AIBD in children. | 4 |
| Lally (2007) | Retrospective study | 101 [17 with dermal-binding LABD and 83 with non dermal-binding LABD] | Patients with dermal-binding LABD resulted clinically indistinguishable from those with non dermal-binding LABD, and showed no evidence of the classic mechanobullous epidermolysis bullosa acquisita phenotype | 41 |
| Leonard (1982) | Prospective study | 42 [34 with linear deposits and 8 with linear-granular deposits] 35 patients with dermatitis herpetiformis served as controls | A female predominance in the group with linear deposits, while a male predominance was observed in the group with linear-granular deposits. in the group with linear deposits, some patients had a rash typical of dermatitis herpetiformis whilst others resembled pemphigoid. The linear-granular group clinically resembled the dermatitis herpetiformis group | 30 |
| Lings (2015) | Retrospective study | 23 [16 adults and 7 children] | LABD has a biphasic course. Dapsone was the first-line treatment. | 34 |
| Nanda (2006) | Retrospective study | 8 [children] | Four patients (50%) presented an association with either an autoimmune disease or an infection. Dapsone alone or in combination with systemic steroids was the first-line treatment. Seventy-one percent of patients achieved complete remission by the end of 2 years. | 7 |
| Ohata (2017) | Retrospective study | 101 | Autoantigens of LABD are heterogeneous. In a cohort of LABD patients with only IgA deposits along the BMZ on DIF, 53% had IgG autoantibodies detected by means of IIF, immunoblotting or ELISA. Clinical manifestations were similar in patients with and without IgG autoantibodies. | 3 |
| Paige (1997) | Retrospective study | 70 [adults] | Five patients (7.1%) were affected by ulcerative colitis. The pathogenetic mechanisms underlying this association are unclear, albeit abnormalities in colonic mucosal B cells and mucosal IgA1 production reported in patients with ulcerative colitis may play a role in the development of the disease. | 37 |
| Willsteed (1990) | Prospective study | 63 [27 adults and 36 children] | IIF on human skin was positive for IgA in just over 80% of children and approximately 40% of adults and salt-split skin technique demonstrated an increase in the sensitivity of IIF. | 40 |
| Wojnarowska (1988) | Prospective study | 50 [25 adults and 25 children] | Clinical features were similar in adults and children, with mucosal involvement being present in 80% of adults and 64% of children. Remission occurred in 64% of children and 48% of adults. Adult-onset and childhood-onset LABD are two variants of the same disease | 6 |

*Abbreviations: AIBD: autoimmune bullous disease; BMZ: basement membrane zone: DIF: direct immunofluorescence; ELISA: enzyme-linked immunoassay; Ig: immunoglobulin; IIF: indirect immunofluorescence; LABD: linear IgA bullous dermatosis; Ref.: reference*
