# Supplementary material for: Linear IgA bullous dermatosis in adults and children: a clinical and immunopathological study of 38 patients
Source: Orphanet J Rare Dis. 2019 May 24;14:115. doi: 10.1186/s13023-019-1089-2 (PMC6534856; doi:10.1186/s13023-019-1089-2)
Supplement: Supplementary file 2 — Table S2. Summary of the main studies on linear IgA bullous dermatosis including study design, patients’ number, clinical features and laboratory findings. (DOCX 25 kb) [file 13023_2019_1089_MOESM2_ESM.docx]

ADDITIONAL FILE

TABLE S1. Demographic data, clinical features, diagnostic findings, treatments and outcomes of 38 linear IgA bullous dermatosis patients

| **Patient N°** | **Age (yrs) / sex** | **Comorbidities** | **Histology** | **Clinical features** | **DIF** | **Salt-split skin IIF** | **Treatment** | **Response (follow-up time, months)** | **Drug-induction (NS)** | **Pre-biopsy diagnosis** |
| --- | --- | --- | --- | --- | --- | --- | --- | --- | --- | --- |
| 1 | F/34 | None | Subepidermal blister, neutrophils, eosinophils | Vesiculobullous eruption/SOF/inframammary and retroauricular folds and scalp | Linear IgA and C3c deposits along the BMZ | Linear IgA deposits (epidermal side of the BMZ) | Dapsone + intravenous methylprednisolone, plasmapheresis, cyclophosphamide, mycophenolate mofetil, rituximab | NR (132) | None | LABD |
| 2 | M/29 | None | Subepidermal blister, neutrophils | Vesiculobullous eruption/periumbilical region, perioral area | Linear IgA deposits along the BMZ | Negative | Dapsone | CR (37) | None | LABD |
| 3 | F/74 | OA | Subepidermal blister, neutrophils | Erosive-crusted and excoriated lesions/trunk | Linear IgA deposits along the BMZ | Negative | Prednisone, methotrexate + oral methylprednisolone | CR (97), relapse at month 52 | None | BP |
| 4 | F/35 | None | Subepidermal blister, neutrophils, eosinophils | Blisters/SOF/trunk, upper limbs | Linear IgA deposits along the BMZ | Negative | Dapsone | CR (35) | None | Pemphigus herpetiformis |
| 5 | M/72 | None | Subepidermal blister, neutrophils, eosinophils | Blisters/scalp, upper limbs | Linear IgA and granular C3c deposits along the BMZ | ND | Dapsone + prednisone | CR (62), relapse at month 41 | None | EBA |
| 6 | F/80 | CLL | Subepidermal blister, neutrophils, eosinophils | Blisters/trunk, upper and lower limbs | Linear/granular IgA deposits along the BMZ | Negative | Deflazacort + topical clobetasol | CR (4) | Chlorambucil (2) | BP |
| 7 | F/51 | None | Subepidermal blister, neutrophils, eosinophils | Blisters/inframammary folds | Linear IgA deposits along the BMZ | Linear IgA deposits (epidermal side of the BMZ) | Dapsone + azathioprine + prednisone | CR (18) | None | BP |
| 8 | M/75 | AH, CAD, CKD | Subepidermal blister, neutrophils | Blisters and erosions/ upper limbs, buttocks; erosions/oral cavity | Linear/granular IgA and granular C3c deposits along the BMZ | ND | Dapsone, prednisone | CR (8) | None | BP |
| 9 | M/68 | CD, nodular prurigo | Subepidermal blister, neutrophils, eosinophils | Urticarial lesions/elbows, knees, popliteal fossae, inguinal folds | Linear/granular IgA deposits and C3c cytoid bodies along the BMZ | Linear IgA deposits (epidermal side of the BMZ) | Dapsone, oral methylprednisolone | CR (5) | None | DH |
| 10 | F/41 | None | Subepidermal blister, neutrophils, eosinophils | Vesiculobullous eruption/ upper limbs | Linear/granular IgA, C3c and IgM deposits along the BMZ; | Negative | Prednisone | CR (69) | None | Bullous insect bite reaction |
| 11 | F/75 | None | Subepidermal blister, neutrophils, eosinophils | Vesiculobullous eruption/trunk, upper limbs | Linear IgA deposits along the BMZ | Negative | Prednisone | CR (10) | None | LABD |
| 12 | M/53 | GERD, IV  AH, DVT | Subepidermal blister, neutrophils, eosinophils | Blisters/upper limbs, inguinal folds, trunk, buttocks; erosions/oral cavity | Linear/granular IgA deposits along the BMZ | ND | Dapsone + prednisone, methotrexate | CR (71), relapse at month 45 | None | LABD |
| 13 | M/54 | None | Subepidermal blister, neutrophils | Vesiculobullous, erosive and excoriated lesions/upper and lower limbs | Linear IgA deposits along the BMZ | Negative | Prednisone, dapsone + topical methylprednisolone | CR (50) | None | BP vs eczema |
| 14 | F/64 | None | Subepidermal blister, neutrophils, eosinophils | Blisters/upper and lower limbs; erosions and symblepharon/ corneal-conjunctival surface of both eyes; erosions/gingival mucosa | Linear IgA deposits along the BMZ | Linear IgA deposits (epidermal side of the BMZ) | Dapsone + prednisone; cyclosporine eye drops | PR (62) | None | CP |
| 15 | F/67 | AD, epilepsy, AH | Subepidermal blister, neutrophils | Vesiculobullous eruption/ SOF/ scalp trunk, buttocks | Linear IgA deposits along the BMZ | Negative | Dapsone + prednisone + topical clobetasol | CR (20) | Oxcarbazepine (3) | BP |
| 16 | F/66 | AH, CAD | Subepidermal blister, neutrophils, eosinophils | Vesicles on erythematous base/trunk, lower limbs | Linear IgA and linear/granular C3c deposits along the BMZ | Linear IgA deposits (epidermal side of the BMZ) | Dapsone + prednisone | CR (41), relapse at month 33 | None | Eczema |
| 17 | F/69 | None | Subepidermal blister, neutrophils | Blisters/trunk | Linear IgA and C3c and linear/granular IgM deposits along the BMZ | ND | Dapsone, prednisone | CR (39) | None | Herpes zoster, pemphigus vulgaris |
| 18 | M/51 | UC, osteoporosis | Subepidermal blister, neutrophils, eosinophils | Erosions and crusts/upper and lower limbs, trunk, scalp; blisters/trunk, lower limbs | Linear IgA deposits along the BMZ | Linear IgA deposits (epidermal side of the BMZ) | Dapsone + prednisone, azathioprine, cyclophosphamide, mycophenolate mofetil, rituximab, methotrexate | NR (38) | None | Eczema |
| 19 | F/61 | None | Subepidermal blister, neutrophils | Vesicles and crusted lesions/ upper and lower limbs | Linear IgA deposits along the BMZ | Linear IgA deposits (epidermal side of the BMZ) | Azathioprine + prednisone + topical clobetasol, dapsone | CR (36) | None | Herpes zoster |
| 20 | F/82 | AH, GERD | Subepidermal blister, neutrophils, eosinophils | Blisters/lower limbs | Linear IgA deposits along the BMZ | Linear IgA deposits (epidermal side of the BMZ) | Dapsone | N/A | None | BP |
| 21 | F/71 | Breast cancer | Subepidermal blister, neutrophils | Urticarial lesions and blisters/upper limbs (>> back of the hands) | Linear/granular IgA deposits along the BMZ | Linear IgA deposits (epidermal side of the BMZ) | Dapsone | CR (27) | None | Chronic urticaria |
| 22 | M/43 | Renal transplant for CKD, β-thalassemia minor | Subepidermal blister, neutrophils, eosinophils | Vesiculobullous lesions/trunk; crusted lesions on erythematous base/ face (beard area) | Linear IgA deposits along the BMZ | Linear IgA deposits (epidermal side of the BMZ) | Prednisone, dapsone + topical clobetasol | CR (13) | None | Sycosis vulgaris |
| 23 | M/93 | AH, ischemic stroke | Subepidermal blister, neutrophils, eosinophils | Blisters on erythematous base/ trunk, upper and lower limbs | Linear IgA deposits along the BMZ; granular IgA perivascular deposits | Linear IgA deposits (epidermal side of the BMZ) | Prednisone | CR (5) | Amoxicillin/clavulanic acid (6) | BP |
| 24 | M/81 | Prostate cancer, AH, chronic HCV infection | Subepidermal blister, neutrophils, eosinophils | Blisters on urticarial base/lower and upper limbs, buttocks | Linear IgA deposits along the BMZ | Negative | Dapsone + prednisone | CR (17) | Losartan (2) | Erythema multiforme vs chronic urticaria |
| 25 | M/46 | None | Subepidermal blister, neutrophils | Blisters/trunk, upper and lower limbs | Linear IgA deposits along the BMZ | Linear IgA deposits (epidermal side of the BMZ) | Prednisone | N/A | None | BP |
| 26 | M/76 | MEN 1 | Subepidermal blister, neutrophils | Blisters/upper and lower limbs; erosions and ulcerations/oral cavity, conjunctiva of both eyes | Linear IgA deposits along the BMZ | Linear IgA deposits (epidermal side of the BMZ) | Rituximab + intravenous methylprednisolone + dapsone, cyclosporine eye drops | PR (17) | None | CP |
| 27 | M/26 | None | Subepidermal blister, neutrophils, eosinophils | Vesiculobullous eruption/trunk, ankles | Linear IgA deposits along the BMZ | Negative | Self-resolution after drug withdrawal | CR (16) | Clarithromycin (5) | BP |
| 28 | F/6 | None | Subepidermal blister, neutrophils, eosinophils | Vesiculobullous eruption on erythematous skin/ trunk, ears, lower and upper limbs; vesicles and erosions/genital mucosa, | Linear IgA deposits along the BMZ | ND | Dapsone + oral betamethasone | CR (12) | None | Bullous impetigo |
| 29 | M/7 | None | Subepidermal blister, neutrophils | Blisters/face (>> perioral area), oral cavity, nasal cavity | Linear IgA deposits along the BMZ | Linear IgA deposits (epidermal side of the BMZ) | Self-resolution without therapy | CR (16) | None | Herpes simplex virus infection |
| 30 | F/4 | None | Subepidermal blister, neutrophils, eosinophils | Vesicles on erythematous skin/perioral and periumbilical area; blisters/trunk; erosions and vesicles/genital mucosa, perianal area | Linear IgA deposits along the BMZ | ND | Dapsone | CR (6) | None | Atopic dermatitis |
| 31 | M/0.9 | None | Subepidermal blister, neutrophils | Vesicles on erythematous skin/ face, trunk | Linear/granular IgA and linear IgM and C3c deposits along the BMZ | Linear IgA deposits (epidermal side of the BMZ) | Dapsone + prednisone | CR (5) | None | LABD |
| 32 | M/14 | UC | Subepidermal blister, neutrophils | Blisters/trunk, upper and lower limbs | Linear IgA deposits along the BMZ | Linear IgA deposits (epidermal side of the BMZ) | Dapsone + prednisone | CR (8) | None | Chickenpox |
| 33 | M/3 | None | Subepidermal blister, neutrophils, eosinophils | Blisters/SOF/lower and upper limbs, face (>> perioral area) | Linear IgA deposits and IgM cytoid bodies along the BMZ; granular C3c deposits at dermal tips | Linear IgA deposits (epidermal side of the BMZ) | Dapsone | CR (22) | None | Bullous impetigo |
| 34 | M/2 | None | Subepidermal blister, neutrophils | Vesiculobullous eruption/SOF/perioral area, upper limbs, ears; erosions/genital mucosa | Linear IgA deposits along the BMZ | ND | Dapsone | CR (19) | None | Bullous impetigo |
| 35 | F/10 | None | Subepidermal blister, neutrophils | Vesicles and crusted lesions on erythematous skin/trunk, lower limbs; ulceration/oral cavity | Linear/granular IgA, IgG and C3c deposits along the BMZ | ND | Dapsone, prednisone | PR (26) | None | Bullous impetigo vs ACD |
| 36 | M/1 | None | Subepidermal blister, neutrophils, eosinophils | Vesiculobullous eruption on erythematous skin/SOF/ lower and upper limbs, face (>> perioral area) | Linear IgA deposits and IgM cytoid bodies along the BMZ | Linear IgA deposits (epidermal side of the BMZ) | Dapsone | CR (32), relapse at month 26 | None | LABD |
| 37 | M/4 | None | Subepidermal blister, neutrophils, eosinophils | Blisters/SOF/ face (>>perioral area), ears, lower limbs, elbows, palmoplantar regions, buttocks | Linear IgA, C3c, IgG and IgM deposits along the MBZ | Negative | Dapsone + prednisone | CR (25) | None | Bullous impetigo |
| 38 | M/3 | None | Subepidermal blister, neutrophils | Blisters/SOF/Back of the hands, lower limbs, trunk | Linear IgA deposits along the BMZ | Linear IgA deposits (epidermal side of the BMZ) | Dapsone + prednisone + topical gentamycin/  betamethasone | CR (28) | None | LABD |

ACD, allergic contact dermatitis; AD, Alzheimer’s disease; AH, arterial hypertension; BP, bullous pemphigoid; BMZ basement membrane zone; C3c, complement fraction 3; CAD, coronary artery disease; chronic lymphatic leukemia; CKD, chronic kidney disease; CP, cicatricial pemphigoid; CR, complete remission; DH, dermatitis herpetiformis; DVT, deep vein thrombosis; EBA, epidermolysis bullosa acquisita; GERD, gastroesophageal reflux disease; IV, ichthyosis vulgaris; MEN1, multiple endocrine neoplasia type 1; N/A, not available; ND, not done; NR, no response; NS, Naranjo score; OA, osteoarthrosis; PR, partial remission; SOF, “string of pearls” pattern; UC, ulcerative colitis
